# Grape seed proanthocyanidin extract targets p66Shc to regulate mitochondrial biogenesis and dynamics in diabetic kidney disease

**DOI:** 10.3389/fphar.2022.1035755

**Published:** 2023-01-06

**Authors:** Yiyun Song, Hui Yu, Qiaoling Sun, Fei Pei, Qing Xia, Zhaoli Gao, Xianhua Li

**Affiliations:** ^1^ Department of Nephrology, Qilu Hospital of Shandong University, Jinan, Shandong, China; ^2^ Cheeloo College of Medicine, Shandong University, Jinan, Shandong, China; ^3^ Department of Nephrology, Qilu Hospital of Shandong University (Qingdao), Qingdao, Shandong, China

**Keywords:** diabetic kidney disease, grape seed proanthocyanidin extract, mitochondrial biogenesis, mitochondrial dynamics, p66Shc

## Abstract

Mitochondrial biogenesis and dynamics are associated with renal mitochondrial dysfunction and the pathophysiological development of diabetic kidney disease (DKD). Decreased p66Shc expression prevents DKD progression by significantly regulating mitochondrial function. Grape seed proanthocyanidin extract (GSPE) is a potential therapeutic medicine for multiple kinds of diseases. The effect of GSPE on the mitochondrial function and p66Shc in DKD has not been elucidated. Hence, we decided to identify p66Shc as a therapeutic target candidate to probe whether GSPE has a renal protective effect in DKD and explored the underlying mechanisms. Methods. *In vivo*, rats were intraperitoneally injected with streptozotocin (STZ) and treated with GSPE. Biochemical changes, mitochondrial morphology, the ultrastructure of nephrons, and protein expression of mitochondrial biogenesis (SIRT1, PGC-1α, NRF1, TFAM) and dynamics (DRP1, MFN1) were determined. *In vitro*, HK-2 cells were transfected with p66Shc and treated with GSPE to evaluate changes in cell apoptosis, reactive oxygen species (ROS), mitochondrial quality, the protein expression. Results. *In vivo*, GSPE significantly improved the renal function of rats, with less proteinuria and a lower apoptosis rate in the injured renal tissue. Besides, GSPE treatment increased SIRT1, PGC-1α, NRF1, TFAM, and MFN1 expression, decreased p66Shc and DRP1 expression. *In vitro*, overexpression of p66Shc decreased the resistance of HK-2 cells to high glucose toxicity, as shown by increased apoptosis and ROS production, decreased mitochondrial quality and mitochondrial biogenesis, and disturbed mitochondrial dynamic homeostasis, ultimately leading to mitochondrial dysfunction. While GSPE treatment reduced p66Shc expression and reversed these changes. Conclusion. GSPE can maintain the balance between mitochondrial biogenesis and dynamics by negatively regulating p66Shc expression.

## 1 Introduction

Diabetic kidney disease (DKD), as the major microvascular complication of diabetes, has become the leading cause of end-stage renal disease worldwide. In recent years, although great progress has been made in the clinical therapies of diabetes, the process of DKD is still uncontrollable (Lytvyn et al., 2020). Thus, further study of its pathogenesis and search for valid therapeutic targets are crucial for the prognosis of DKD.

The conventional perspective of the pathology of DKD emphasized that podocyte injury is often the first target of hyperglycemic damage (Lassen and Daehn, 2020). Recently, renal tubular epithelial cell apoptosis and tubular atrophy have been recognized as indicators of the severity and progression of DKD (Gilbert, 2017). Numerous studies have found that mitochondrial dysfunction plays a crucial role in the pathobiology of DKD accompanied by renal tubular epithelial cell injury (Xiao et al., 2017; Forbes and Thorburn, 2018; Jiang et al., 2019). Hyperglycemia directly damages mitochondria, resulting in the overproduction of reactive oxygen species (ROS), fragmentation of mitochondria, and reduced efficiency of mitochondrial biogenesis ultimately leading to mitochondrial dysfunction (Galvan et al., 2017). Sirtuin 1(SIRT1)/Peroxisome proliferator-activated receptor-γ coactivator-1α (PGC-1α) pathway and their target genes nuclear respiratory factor (NRF1) and mitochondrial transcription factor A (TFAM) play a critical role in mitochondrial biogenesis (Yacoub et al., 2014; Xue et al., 2019). Several evidence suggest that increased mitochondrial fission and decreased fusion lead to mitochondrial fragmentation in DKD. Mitofusins 1 (MFN1) and dynamin-related protein 1 (Drp1) have been shown to be major regulators in the maintenance of mitochondrial dynamic homeostasis (Rovira-Llopis et al., 2017).

The 66 kDa Src homology two domain-containing protein (p66Shc) is a recognized intracellular critical factor that participates in regulating aging and metabolic disorders (Mir et al., 2020). In the recent past, p66Sch has been reported to be a novel renal marker that plays a significant role in the development of DKD (Xu et al., 2016). Recent studies have illustrated that the expression of p66Shc is increased in podocytes of the kidney of diabetic patients (Zheng et al., 2020). Under hyperglycemia conditions, p66Shc is phosphorylated at Ser36 and translocated into mitochondria, releasing cytochrome c and disrupting the electron transport chain (Giorgio et al., 2005; Mir et al., 2020). Besides, p66Shc can affect mitochondrial dynamics while participating in mitochondrial biogenesis by regulating SIRT1 expression (Qu et al., 2018; Wang et al., 2020). These events generate large quantities of ROS, reduce mitochondrial quality and activate apoptotic mechanisms (Giorgio et al., 2005). Furthermore, the genetic deletion of p66Shc significantly attenuated renal oxidative stress and pathological lesions and safely protected the kidneys of DKD (Menini et al., 2006; Sun et al., 2010). Hence, inhibition of p66Shc expression is a promising approach for the treatment of DKD.

Grape seed proanthocyanidin extract (GSPE) is an effective natural plant polyphenolic antioxidant (Prasain et al., 2009), which exhibits lots of effects, such as anti-inflammatory, antioxidant, and antitumor activities in a variety of diseases (Zhan et al., 2016; Serrano et al., 2017; Hao et al., 2018; Yang et al., 2018). Our previous study demonstrated that GSPE reduced proteinuria and attenuated endoplasmic reticulum stress in diabetic rats (Li et al., 2017; Gao et al., 2018). Thus, we propose that GSPE may serve as a potential therapeutic medicine in protecting the kidney from hyperglycemic toxicity. However, there are few studies on the effect of GSPE on p66Shc in DKD, and convincing studies are needed. Therefore, we identified p66Shc as a therapeutic candidate target to probe whether GSPE has a renoprotective effect in DKD and to explore the underlying mechanisms.

## 2 Materials and methods

### 2.1 Experimental animal

40 male Sprague-Dawley (SD, 190 ± 10g, 7 weeks old) rats were obtained from Shandong University Animal Experiment Center (Jinan, China). The rats were housed with a 12 h light/dark cycle and free access to food and water at a temperature of 20°C–25°C and humidity of 40–60%. The rats were randomly divided into four groups: control group, control + GSPE group, diabetic model (DM) group, and DM + GSPE group. Diabetic rats were induced with SD rats *via* a single intraperitoneal injection of 40 mg/kg streptozotocin (STZ), freshly dissolved in 0.1 mol/L citrate buffer (pH 4.3). The control animals were given a single intraperitoneal injection with an equal volume of citrate buffer. Rats with blood glucose levels ≥16.7 mmol/L were successfully modeled for diabetes. According to the report, GSPE at a concentration of 250 mg/kg exhibited the most potent renoprotection (Zhan et al., 2016). Therefore, we choose this concentration for the following experiments. After diabetic model formation, rats in the DM + GSPE group and the control + GSPE group were given 250 mg/kg/d GSPE by intragastric administration and maintained for 12 weeks. The control group and DM group were filled with the same amount of physiological saline. Rats in the control group and the control + GSPE group were fed a normal diet. At the same time, the remaining rats in the DM group and the DM + GSPE group were fed a high-sucrose-high-fat diet. At the end of the experiment, all 10 rats survived in the control group and control + GSPE group, six rats survived in the DM group, and seven in the DM + GSPE group. All experiments involving animals were conducted in strict accordance with the procedure which was approved by the Institutional Animal Care and Use Committee of Shandong University.

### 2.2 Metabolic Measurements

Metabolic Measurements: The rats’ body weights and random blood glucose levels were measured at the end of the experiment. After 12 weeks of treatment, the rat’s urine was collected for urinary albumin analysis. At the time that the rats were sacrificed, blood and tissue samples were harvested and processed for various studies. Metabolic conditions were measured using an automatic biochemical analyzer (Hitachi AutoAnalyzer 7100, Hitachi, Japan).

### 2.3 Assessment of renal tissue morphology and transmission electron microscopy

The kidneys were excised for histological analysis. The sections of renal biopsy from rats were stained with Hematoxylin and Eosin (HE) or periodic acid Schiff (PAS) staining. According to the methods from the literature (Lu et al., 2017), the degree of damage in each glomerulus was assessed using a semiquantitative scoring method. The glomerular matrix expansion index (GMI) was then calculated. The ultrastructure of podocytes and mitochondria in renal tissue was observed using transmission electron microscopy (TEM).

### 2.4 TUNEL staining

Apoptosis was detected with a Terminal deoxynucleotidyl transferase dUTP nick-end labeling (TUNEL) kit (11684817910, Roche, Switzerland), according to the manufacturer’s instructions. Nuclei were visualized by staining with DAPI for 5 min at room temperature. Digital images were captured using a fluorescence microscope (Nikon Eclipse C1, Nikon, Japan). The percentage of the positive cells was analyzed by ImageJ software.

### 2.5 Immunohistochemistry

The expression of SIRT1, PGC-1α, NRF1, TFAM, Cleaved caspase-3, MFN1, DRP1, and p66Shc in kidney tissues of different groups was detected by immunohistochemistry analysis. The average optical density (AOD) was quantified with ImageJ software.

### 2.6 Western blotting assay

Renal tissues and cells were lysed using RIPA lysis fluid. The protein concentration was determined using a BCA protein assay kit (P0010S, Beyotime, China). Equal amounts of protein were subjected to electrophoresis on sodium dodecyl sulfate-polyacrylamide gels (SDS-PAGE) and then transferred to polyvinylidene difluoride (PVDF) membranes. The membranes were blocked with 5% nonfat milk and incubated with one of the following primary antibodies: anti-Cleaved caspase-3 (9661/R, 1:1000, Cell Signaling Technology, United States), anti-SIRT1(ab189494, 1:1000, Abcam, UK), anti-PGC-1α(ab191838, 1:1000, Abcam, UK), anti-NRF1(ab175932, 1:1000, Abcam, UK), anti-TFAM(A13552, 1:1000, Abclonal, China), anti-MFN1(13798-1-AP, 1:1000, Proteintech, China), anti-DRP1 (ab184247, 1:1000, Abcam, UK), anti-p66Shc (ab33770, 1:1000, Abcam, UK), anti-cytochrome C(CytoC) (ab133504, 1:5000, Abcam, UK), anti- DIABLO (ab32023, 1:1000, Abcam, UK), overnight at 4°C. After washing with TBST, the PVDF membranes were incubated with a secondary antibody at 37°C for 1 h. The protein bands on the PVDF membranes were observed.

### 2.7 Cell culture and treatment

**FIGURE 1:**
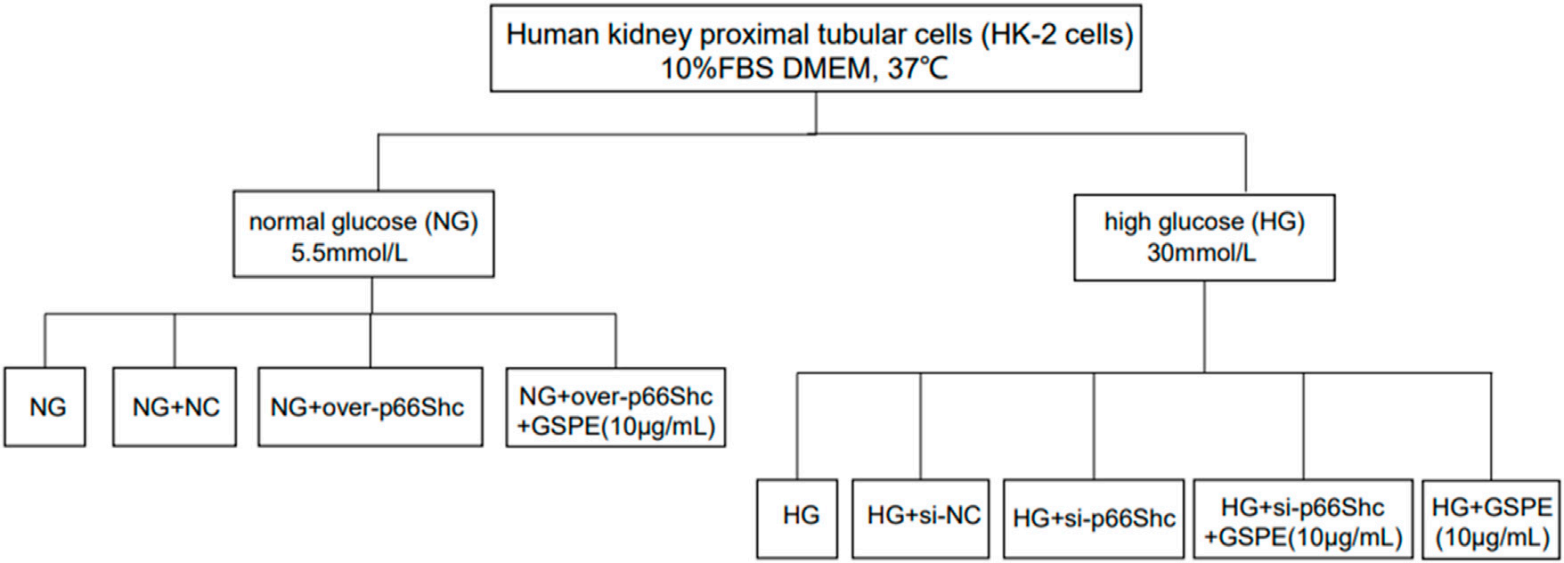
The grouping flow chart.

Human kidney proximal tubular cells (HK-2 cells) were maintained in Dulbecco’s modified Eagle medium (DMEM) containing 10% fetal bovine serum. The cells were placed in a CO_2_ incubator at 37°C. Cells in the logarithmic phase were taken for the subsequent experiment. HK-2 cells were subjected to 5.5 mmol/L glucose as normal glucose (NG) or 30 mmol/L glucose as high glucose (HG) administration. Cells were treated with 10 μg/ml GSPE for 24 h at 37°C according to previous research (Cai et al., 2016). The grouping flow chart is shown in Figure 1 as well.

### 2.8 Transfection

tHK-2 cells were seeded in six-well plates. Cell transfection was performed at a concentration of 100 nmol/L using Lipofectamine 2000 (11668019, Invitrogen, United States) according to the manufacturer’s instructions. HK-2 cells grown in NG were transfected with negative control (NC) plasmid and p66Shc plasmid to overexpression of p66Shc (over-p66Shc) for 48 h. In parallel, cells grown in HG were transfected with siRNA against p66Shc (si-p66Shc), or siRNA negative control (si-NC). After transfection for 48 h, HK-2 cells were then treated with 10 μg/ml GSPE. 24 h after, cells were harvested and utilized for various studies. The efficiency of transfection with p66Shc was determined by western blotting assay and real-time quantitative reverse transcription-polymerase chain reaction (qRT-PCR) (Primer sequences: p66Shc forward primer, ATC​ACT​CTC​ACC​G TC​TCC​ACC​AG; reverse primer, TCTTTGGCAACATAGGC GACATACTC. β-actin forward primer, ACA​CTG​TGC​CCA​TCT​ACG; reverse primer, TGT​CAC​GCA​CGA​TTT​CC).

### 2.9 Measurement of apoptosis and superoxide generation

The commonly used apoptosis kit (AP105, MultiSciences, China) was performed for apoptosis assessment. The ratio of apoptotic cells was assessed by flow cytometry. Mitochondrial superoxide generation was detected using MitoSOX red mitochondrial superoxide indicator (40778ES50, Yeasen, China). 2′,7′-Dichlorodihydrofluorescein diacetate (DCFH-DA, S0033S-1, Beyotime, China) were used to assess intracellular superoxide production in HK-2 cells, respectively.

### 2.10 Measurement of mitochondrial membrane potential (Δψm)

JC-1 probe (C2006, Beyotime, China) was carried out for Δψm detection according to the instruction. The results were assessed by flow cytometry.

### 2.11 Measurement of the activity of mitochondrial respiratory chain enzyme complexes Ⅰ and Ⅲ

The activity of complexes Ⅰ and Ⅲ was measured with Micro Mitochondrial Respiratory Chain Complex Ⅲ Activity Assay Kit (BC3245, Solarbio, China) and Micro Mitochondrial Respiratory Chain Complex Ⅰ Activity Assay Kit (BC0515, Solarbio, China) according to the manufacturer’s instructions.

### 2.12 Statistical analyses

GraphPad Prism 6 (GraphPad Software, Inc., San Diego, United States) was used for statistical analyses. One-way analysis of variance (ANOVA) was employed for comparisons between groups. Post-hoc Tukey’s honestly significant difference test was performed for multiple comparisons. Data were presented as mean ± SD. *p* < 0.05 was considered statistically significant.

## 3 Results

### 3.1 The levels of biochemical indicators in GSPE-treated diabetic rats

Compared with the control group, blood glucose, urinary albumin, and serum creatinine were significantly increased in the DM group, but these indicators were reduced in the DM + GSPE group in comparison with the DM group (*p* < 0.05, Table 1). Meanwhile, compared with the control group, the rats’ body weights were significantly decreased in the DM and control + GSPE groups (*p* < 0.05, Table 1), but left unchanged between DM and DM + GSPE groups (*p* > 0.05, Table 1). Blood glucose levels in the control + GSPE group were lower than in the control group (*p* < 0.05, Table 1). Moreover, urinary albumin and serum creatinine were not significantly different between the control group and the control + GSPE group (*p* > 0.05, Table 1).

**TABLE 1:** Characteristics of the rats at the end of the experiment. Mean ± SD.

	Control n = 10	Control+GSPE n = 10	DM n = 6	DM+GSPE n = 7
Body weight (g)	424.3 ± 28.92	376.6 ± 17.77^a^	321.3 ± 15.22^a^	341.7 ± 18.28
Blood glucose (mmol/L)	8.36 ± 3.6	3.89 ± 2.29^a^	38.95 ± 4.41^a^	27.88 ± 4.26^b^
Serum creatinine (μmol/L)	39.7 ± 2.67	40.9 ± 2.69	57 ± 3.74^a^	44.57 ± 3.69^b^
Urinary albumin (mg/L)	1.23 ± 0.23	1.59 ± 0.94	11.03 ± 3.73^a^	4.18 ± 2.09^b^

^a^ *p* < 0.05 vs. control.

^b^ *p* < 0.05 vs. DM group.

### 3.2 Effects on diabetic renal tissues architecture induced by GSPE

**FIGURE 2:**
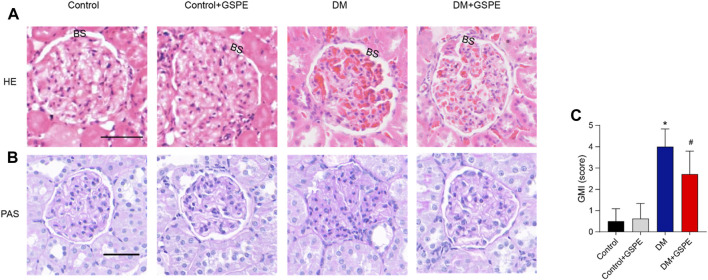
Histopathology evaluation of the kidneys of all rats from all experimental groups as stained by HE and PAS staining. Upper panel **(A)** represented images stained by HE (original magnification, ×200). Lower panel **(B,C)** showed images stained by PAS (Scale bars, 50 μm Original magnification, ×200) and the analysis of the GMI (Mean ± SD. n = 6. ∗*p* < 0.05 vs. control; #*p* < 0.05 vs. DM group). The images of control and control + GSPE-treated rats showed normal kidney structures with normal glomerulus, normal size Bowman’s space (BS). The images of DM rats showed obvious mesangial expansion and glomerulosclerosis. Note the increase in the capsule as well as in Bowman’s space. The images of DM + GSPE rats showed almost normal histological features with the almost normal structure of glomerulus, reduced mesangial expansion and Bowman’s space (BS).

We performed the histological examination of renal tissues from all groups. As shown in HE and PAS staining (Figure 2A), the renal tubules and glomerular structure of the control group and control + GSPE group were natural. The extracellular matrix (ECM), mesangial cell proliferation, Bowman’s space, and glomerulosclerosis increased in the DM group. For GSPE-treated diabetic rats, the situation of these pathological changes was greatly gotten better. The mesangial expansion was assessed by GMI, and the mesangial expansion level in the DM group was significantly larger than that of the control group but inhibited after GSPE treatment (*p* < 0.05, Figure 2B).

### 3.3 Ultrastructural changes of renal tissues in GSPE-treated diabetic rats

**FIGURE 3:**
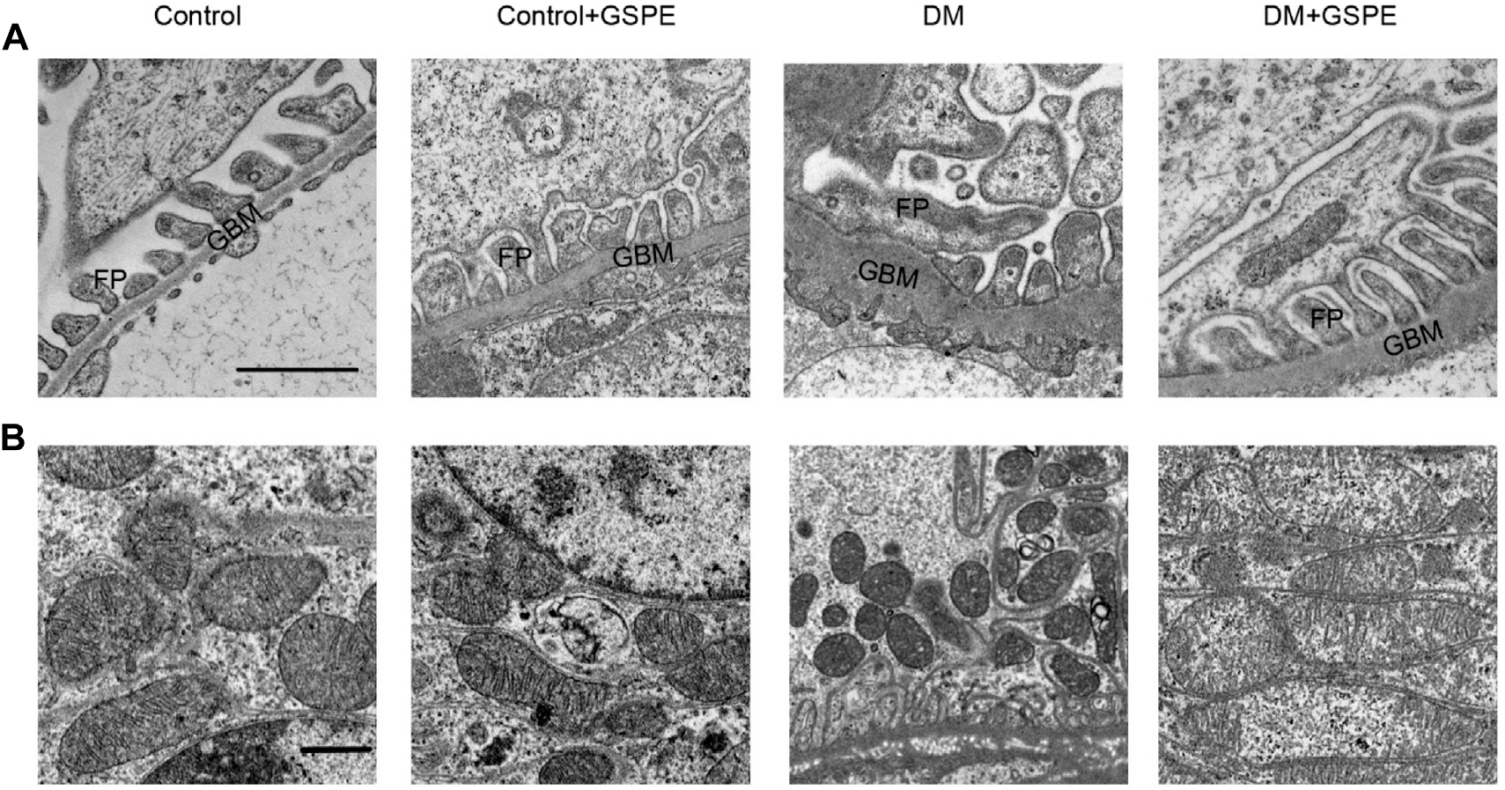
Electron microscopy delineated ultrastructural changes of podocyte and mitochondria in kidney biopsies in each group. **(A)** The images of podocytes were taken by electron microscopy (Scale bars, 1 μm Original magnification, ×10000). **(B)** The images of mitochondria were taken by electron microscopy (Scale bars, 1 μm Original magnification, ×3000). GBM: glomerular basement membrane. FP: foot processes.

The ultrastructural changes in podocytes and mitochondrial morphology were observed through a transmission electron microscope (TEM). As presented in Figure 3, diabetic rats exhibited apparent foot process fusion and glomerular basement membrane (GBM) thickening. However, GSPE treatment considerably reversed these changes in diabetic rats. Elongated rodlike-shaped mitochondria were observed in the control group. The majority of mitochondria were spherical shapes in the DM group. A partial mitochondrial fragmentation was rescued in cells treated with GSPE.

### 3.4 Effect of GSPE on renal cell apoptosis in diabetic rats

**FIGURE 4:**
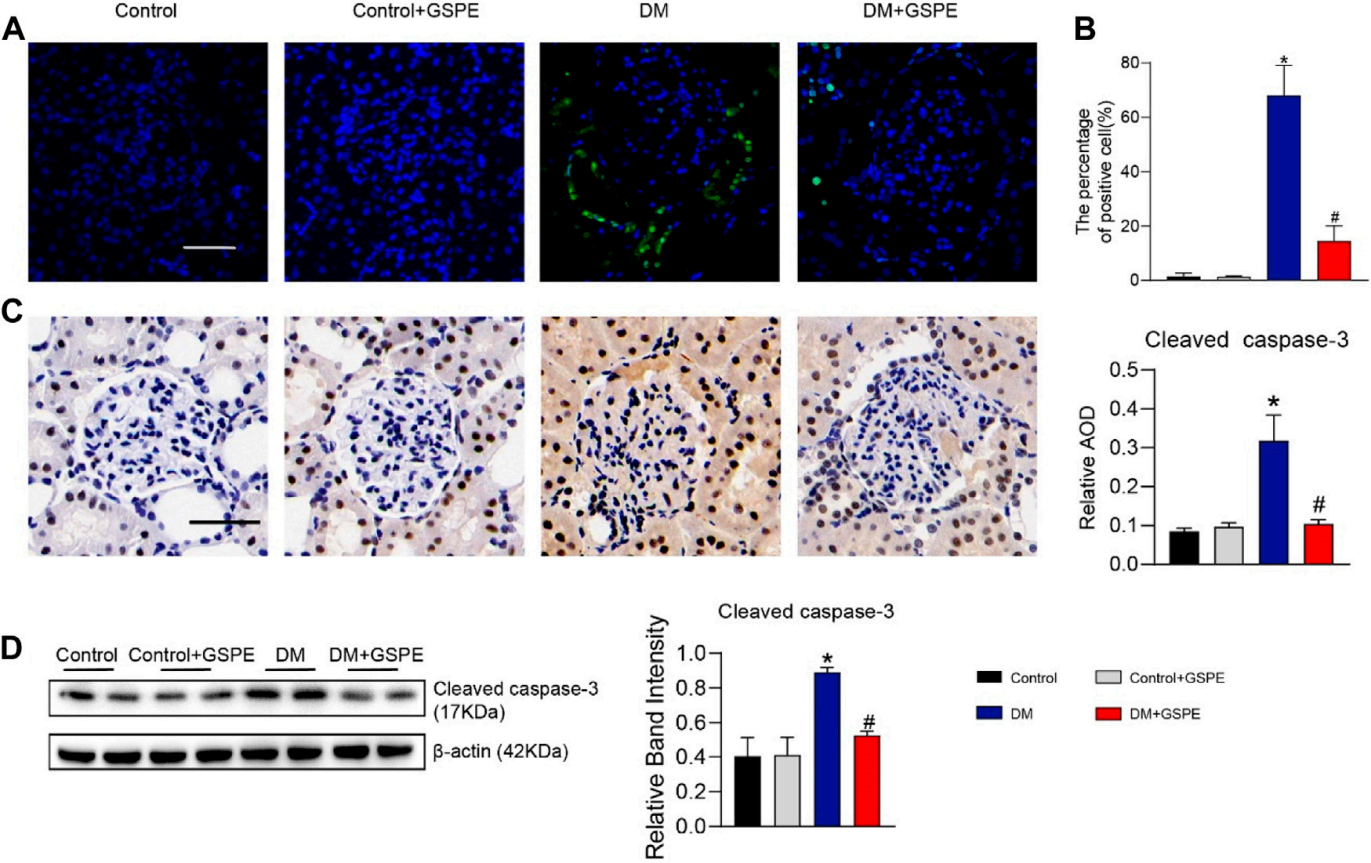
**(A)** The images of cell apoptosis of rat renal tissue. Scale bars, 50 μm. **(B)** Evaluation of apoptosis rate in renal tissue of rats in each group. Mean ± SD. n = 6. ∗*p* < 0.05 vs. control; #*p* < 0.05 vs. DM group. **(C)** Immunohistochemistry delineated the localization and changes in the expression of Cleaved caspase-3 proteins and quantification of relative AOD in each group. (Scale bars, 50 μm Original magnification, ×200) Mean ± SD. n = 6. ∗*p* < 0.05 vs. control; #*p* < 0.05 vs. DM group. **(D)** Western blot for determining the protein levels of Cleaved caspase-3 and quantification of bands in each group. Mean ± SD. n = 6. ∗*p* < 0.05 vs. control; #*p* < 0.05 vs. DM group.

The cell apoptosis of each group was evaluated by TUNEL assays. Compared to the control group, apoptosis was statistically increased in diabetic rats (*p* < 0.05, Figures 4A, B), especially in renal tubular epithelial cells. And GSPE significantly reduced cell apoptosis (*p* < 0.05, Figures 4A, B). Furthermore, western blotting assay and immunohistochemical staining also showed that apoptosis-related protein Cleaved caspase-3 expression was increased in diabetic rats, and GSPE partially inhibited this increase (*p* < 0.05, Figures 4C, D).

### 3.5 Expression of related factors in diabetic rats with GSPE treatment

**FIGURE 5:**
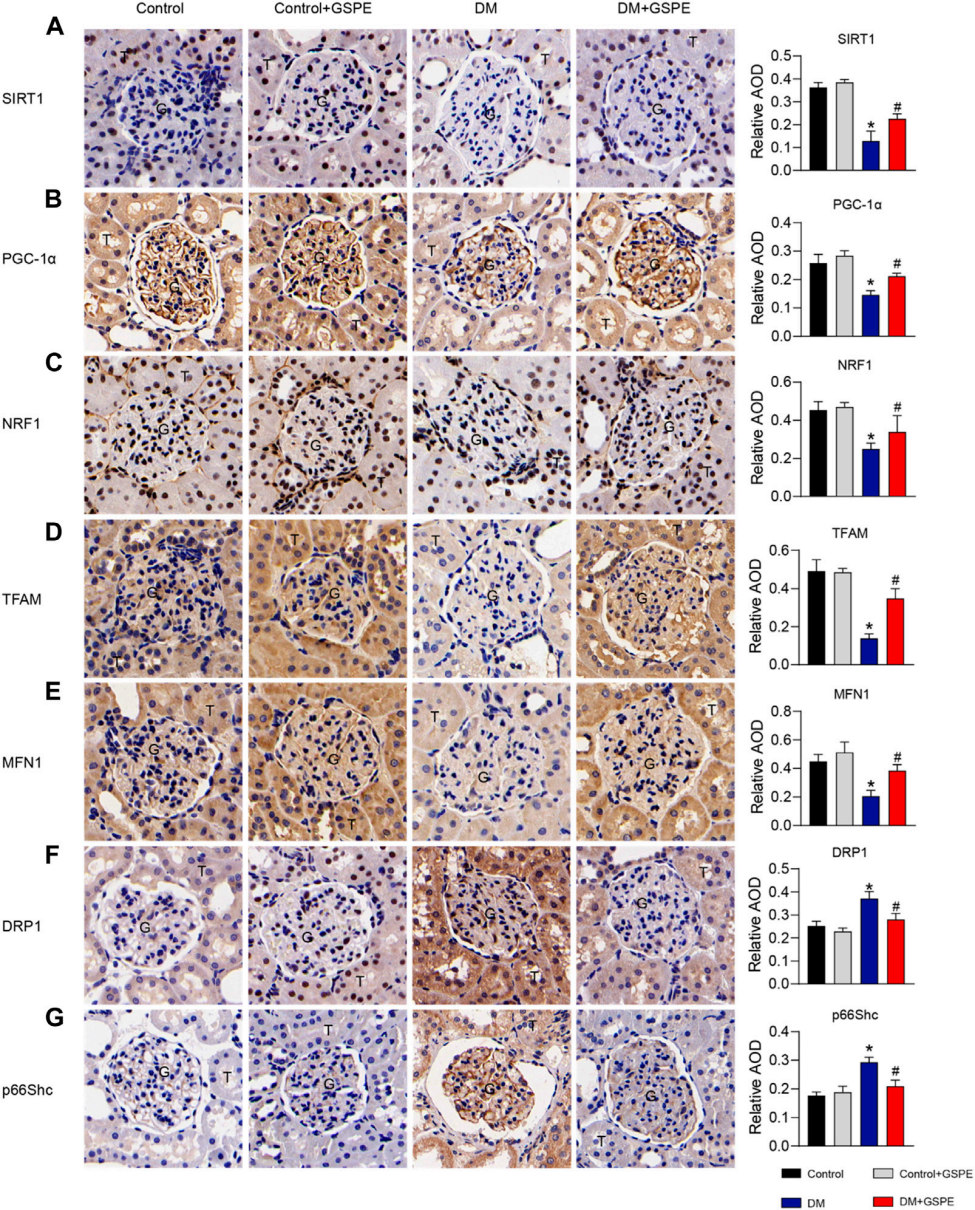
Immunohistochemistry delineated the localization and changes in the expression of proteins and quantification of relative AOD in each group. **(A)** SIRT1 is expressed in the nuclei of renal tubules. **(B)** PGC-1α is expressed in the cytoplasm of renal glomeruli and tubules. **(C)** NRF1 is expressed in the nuclei of renal tubules and partly glomeruli. **(D)** TFAM is expressed in the cytoplasm of renal glomeruli and tubules. **(E)** MFN1 is expressed in the cytoplasm of renal glomeruli and tubules. **(F)** DRP1 is expressed in the cytoplasm of renal tubules and partly glomeruli. **(G)** P66Shc is expressed in the cytoplasm of glomeruli and some tubules. Scale bars: 50 μm. Original magnification, ×200. Mean ± SD. n = 6. ∗*p* < 0.05 vs. control; #*p* < 0.05 vs. DM group. G: glomeruli. T: tubule.

**FIGURE 6:**
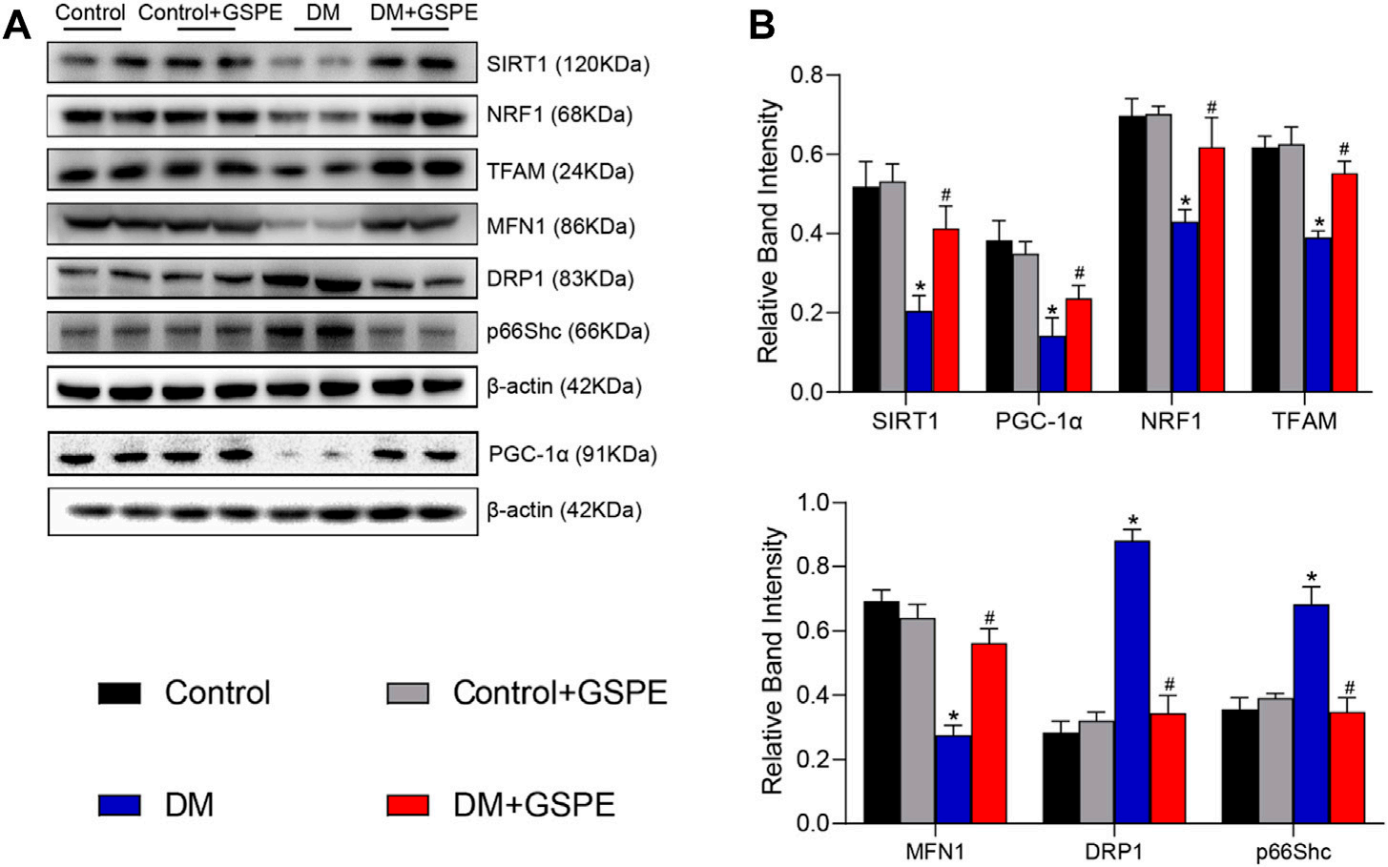
**(A)** Western blot for determining the protein levels of SIRT1, PGC-1α, NRF1, TFAM, MFN1, DRP1 and p66Shc in renal tissue of the rats in each group. **(B)** Quantification of bands in Figure 5A. Mean ± SD. n = 6. ∗*p* < 0.05 vs. control; #*p* < 0.05 vs. DM group.

The role of GSPE in mitochondrial biogenesis and dynamics of DKD progression was verified by immunohistochemical staining and western blotting assay. The results showed that compared with the control group, the relative expression of mitochondrial biogenesis-related proteins (SIRT1, PGC-1α, NRF1, and TFAM) and MFN1 decreased, while Drp1 and p66Shc increased simultaneously in the renal tissue of the DM group (*p* < 0.05, Figures 5A–G). However, the expression of these proteins reversed with the treatment of GSPE (*p* < 0.05, Figures 5A–G). Western blotting analysis was consistent with immunohistochemical results (*p* < 0.05, Figures 6A, B).

### 3.6 The efficiency of transfection was testified

**FIGURE 7:**
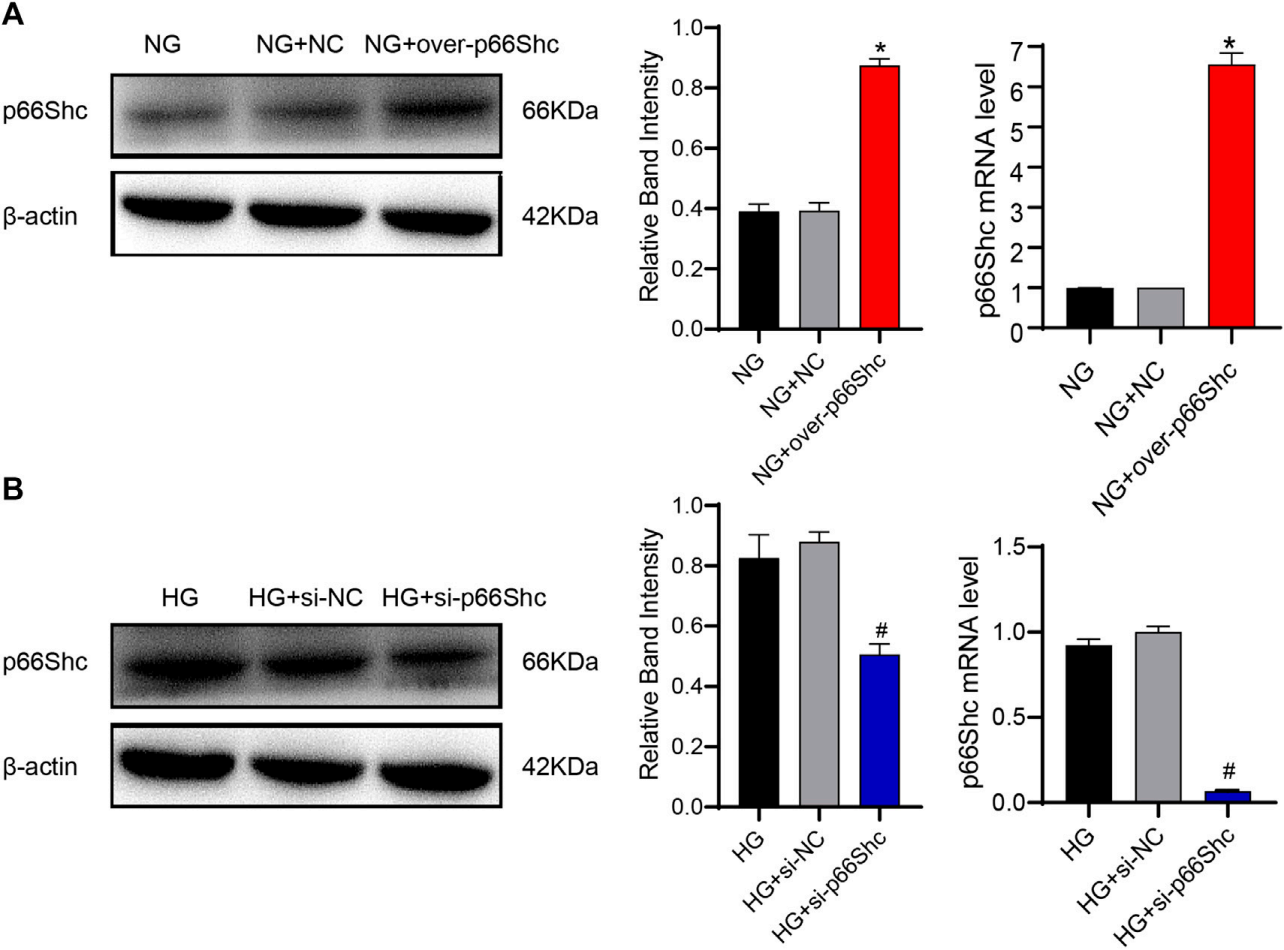
**(A)** Transfection efficiency of over-p66Shc was determined by qRT-PCR and western blotting assay. Mean ± SD. n = 3. ∗*p* < 0.05 vs. NG group. **(B)** Transfection efficiency of si-p66Shc was determined by qRT-PCR and western blotting assay. Mean ± SD. n = 3. #*p* < 0.05 vs. HG group.

To clarify the influence of p66Shc, HK-2 cells were divided into NG, NG + over-p66Shc, NG + NC, HG, HG + si-p66Shc, and HG + si-NC groups. The efficiency of transfection was analyzed by qRT-PCR and western blotting assay. The results showed that the relative expression of p66Shc in the NG + over-p66Shc group was significantly higher (*p* < 0.05, Figure 7A), and that in HG + si-p66Shc group was lower than those in the HG group (*p* < 0.05, Figure 7B). There were no statistically significant differences in mRNA and protein expression between HG and HG + si-NC groups, as well as NG and NG + NC groups (*p* > 0.05, Figures 7A, B), excluding the effect of transfection on cells.

### 3.7 Effect of GSPE on HK-2 cells apoptosis in each group

**FIGURE 8:**
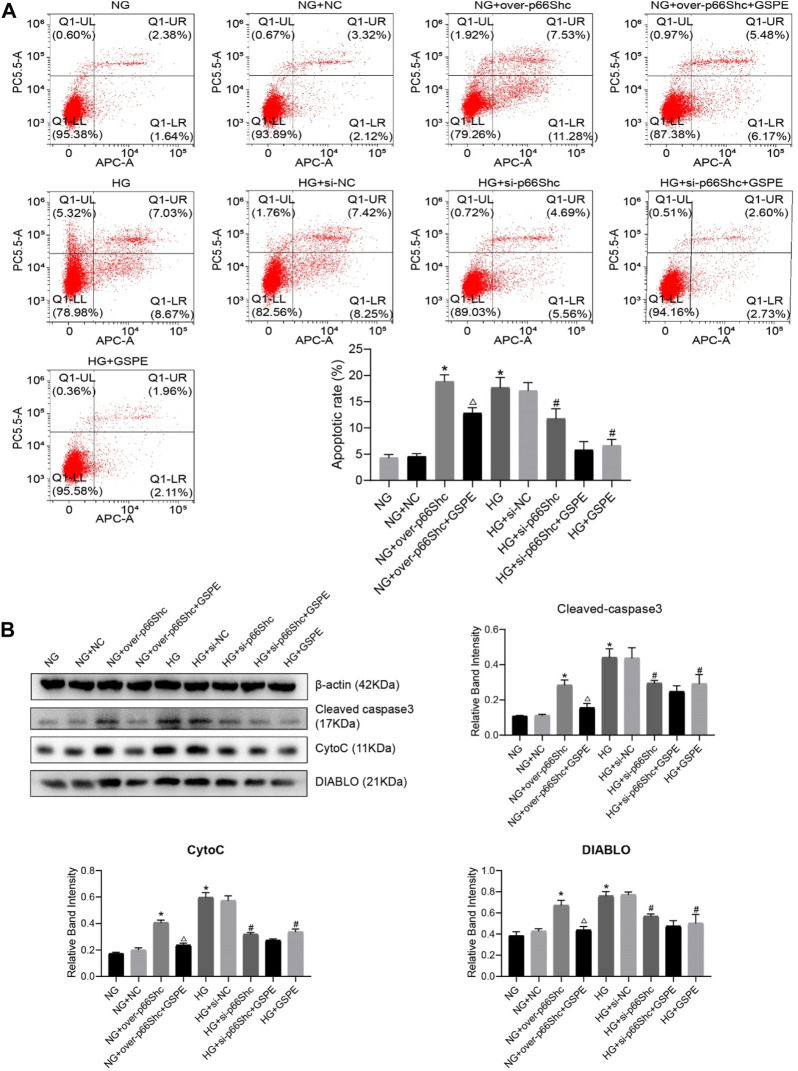
**(A)** Flow cytometry analysis of apoptosis in cultured HK-2 cells in different groups and quantitation of these results. Mean ± SD. n = 3. ∗*p* < 0.05 vs. NG group; #*p* < 0.05 vs. HG group; △*p* < 0.05 vs. NG + over-p66Shc group. **(B)** Western blot for determining the protein levels of mitochondrial associated proteins (Cleaved caspase-3, CytoC, and DIABLO) in each group and quantification of bands. Mean ± SD. n = 3. ∗*p* < 0.05 vs. NG group; #*p* < 0.05 vs. HG group; △*p* < 0.05 vs. NG + over-p66Shc group.

To determine the effect of GSPE, the appropriate GSPE concentration (10 μg/ml) was adopted to treat HK-2 cells. Flow cytometry was performed to detect the apoptotic level. The apoptosis of HK-2 cells significantly increased in HG and NG + over-p66Shc groups as compared with the NG group (*p* < 0.05, Figure 8A). After treatment with GSPE or transfection of si-p66Shc inhibited cell apoptosis (*p* < 0.05, Figure 8A). In addition, the trend of apoptosis-associated proteins Cleaved caspase-3, Cyto C and DIABLO expression is consistent with apoptosis of flow cytometry (*p* < 0.05, Figure 8B).

### 3.8 GSPE alleviates intracellular and mitochondrial ROS generation

**FIGURE 9:**
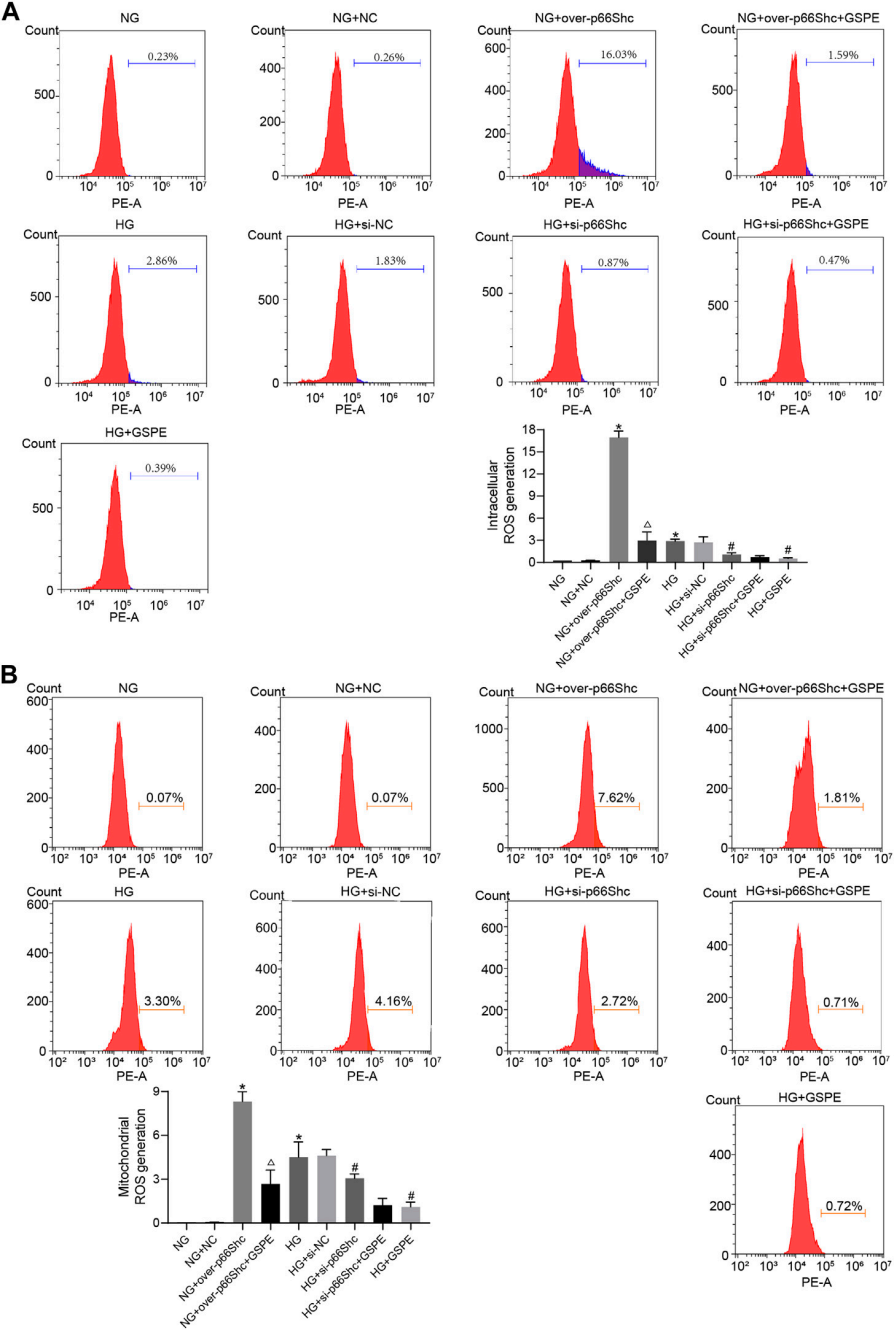
**(A)** Assessment of intracellular ROS generation in various treated groups by DCFH-DA staining. Mean ± SD. n = 3. ∗*p* < 0.05 vs. NG group; #*p* < 0.05 vs. HG group; △*p* < 0.05 vs. NG + over-p66Shc group. **(B)** Assessment of mitochondrial ROS generation in various treated groups by MitoSox staining. Mean ± SD. n = 3. ∗*p* < 0.05 vs. NG group; #*p* < 0.05 vs. HG group; △*p* < 0.05 vs. NG + over-p66Shc group.

DCFH-DA and MitoSOX fluorescent probes were conducted to detect intracellular and mitochondrial oxidative stress. Intracellular and mitochondrial ROS level was markedly increased in HK-2 cells exposed to HG conditions (*p* < 0.05, Figures 9A, B). The level of ROS increased significantly in the NG + over-p66Shc group as compared with the NG group (*p* < 0.05, Figures 9A, B). However, these changes were significantly attenuated by GSPE treatment or transfection of si-p66Shc (*p* < 0.05, Figures 9A, B).

### 3.9 Improvement of mitochondrial quality induced by GSPE

**FIGURE 10:**
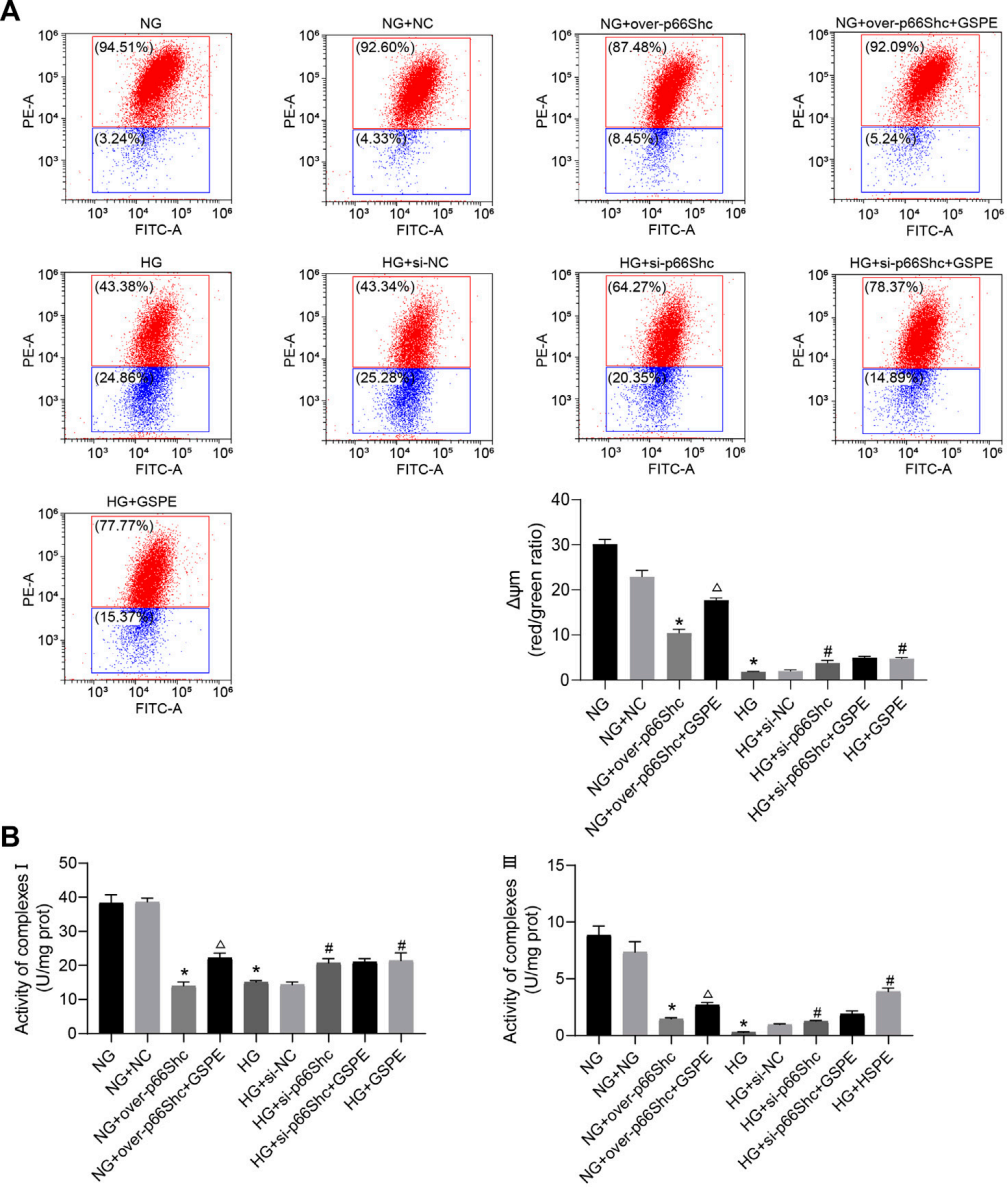
**(A)** Flow cytometry analysis of Δψm in cultured HK-2 cells in different groups and quantitation of these results. Mean ± SD. n = 3. ∗*p* < 0.05 vs. NG group; #*p* < 0.05 vs. HG group; △*p* < 0.05 vs. NG + over-p66Shc group. **(B)** Assessment of the activity of mitochondrial respiratory chain enzyme complexes Ⅰ and Ⅲ. Mean ± SD. n = 3. ∗*p* < 0.05 vs. NG group; #*p* < 0.05 vs. HG group; △*p* < 0.05 vs. NG + over-p66Shc group.

Previous reports have shown that mitochondrial damage is usually accompanied by a decrease in Δψm and the activity of the mitochondrial respiratory chain (Forbes and Thorburn, 2018). We evaluated the transformation of each group on Δψm by JC-1 staining. In healthy mitochondria, JC-1 mainly concentrates as aggregate and emits red fluorescence. In contrast, in mitochondria with reduced Δψm, JC-1 presents mainly in monomeric form and emits green fluorescence. The ratio of red to green fluorescence serves as an indicator of changes in Δψm. The Δψm decreased in HG and NG + over-p66Shc groups, while that was upregulated after si-p66Shc and GSPE treatment (*p* < 0.05, Figure 10A). As shown in Figure 9B, the mitochondrial respiratory chain enzyme complexes Ⅰ and Ⅲ were conspicuously decreased in HK-2 cells by transfection of over-p66Shc or HG treatment. GSPE or transfection of si-p66Shc can increase the activity of mitochondrial respiratory chain enzyme complexes Ⅰ and Ⅲ (*p* < 0.05, Figure 10B).

### 3.10 GSPE exerts kidney protection by inhibiting p66Shc activity in DKD

**FIGURE 11:**
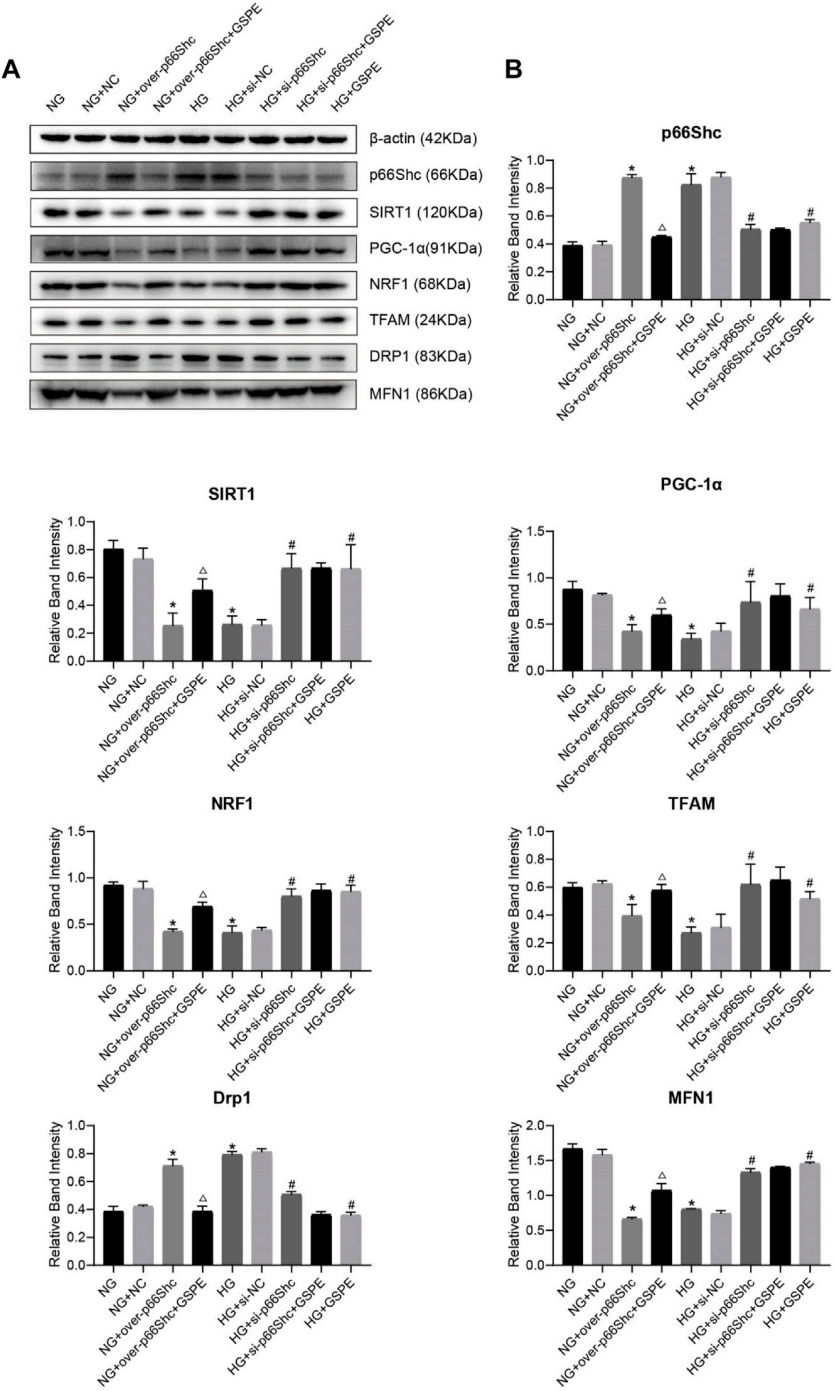
**(A)** Western blot for determining the protein levels of p66Shc, SIRT1, PGC-1α, NRF1, TFAM, MFN1, and DRP1 in each group. **(B)** Quantification of bands in Figure 9A. Mean ± SD. n = 3. ∗*p* < 0.05 vs. NG group; #*p* < 0.05 vs. HG group; △*p* < 0.05 vs. NG + over-p66Shc group.

The effect of GSPE on p66Shc expression in HK-2 cells was evaluated. Western blotting results revealed that p66Shc expression in the NG + over-p66Shc + GSPE group was less than that in the NG + over-p66Shc group (*p* < 0.05, Figures 11A, B). And the expression of p66Shc markedly decreased after GSPE intervention in HK-2 cells exposed to HG conditions (*p* < 0.05, Figures 11A, B), which was consistent with animal experiments.

### 3.11 Expression of related factors in HK-2 cells with GSPE treatment

Western blotting analysis showed that the relative expression of mitochondrial biogenesis-related proteins (SIRT1, PGC-1α, NRF1, and TFAM) and MFN1 were conspicuously decreased after HG treatment as well as transfection of over-p66Shc (*p* < 0.05, Figures 11A, B). While the protein level of Drp1 increased (*p* < 0.05, Figures 11A, B). Whereas p66Shc gene disruption and GSPE treatment reversed the above changes (*p* < 0.05, Figures 11A, B). This coincides with the above results of animal experiments.

## 4 Discussion

DKD is a major microvascular complication in patients with diabetes mellitus and one of the leading causes of end-stage renal disease, contributing to severe morbidity and mortality (Global, 2020). It is characterized by microalbuminuria, glomerulosclerosis, excessive deposition of ECM protein, and GBM thickening, which eventually leads to renal failure (Oshima et al., 2021).

A well-established diabetic rat model with an intraperitoneal injection of STZ partly destroys the function of pancreatic β cells (Lenzen, 2008). Meanwhile, high-sucrose-high-fat diet-induced insulin resistance in rats. High glucose-induced rat models of DKD were established in our present research to investigate the mechanism of GSPE. We could observe hyperglycemia and weight loss in diabetic rats. Urinary albumin and serum creatinine were increased, and glomerular structure was destroyed with obvious ECM and mesangial cell proliferation accompanied by foot process fusion in diabetic rats. These findings indicated the successful rat model of DKD. Moreover, our observation demonstrated that apoptosis of renal tubules was much more than that of glomeruli in diabetic rats, which confirmed the important role of tubular injury in the early stage of DKD. GSPE was demonstrated to decrease blood glucose, relieve renal dysfunction and proteinuria, and alleviate renal damage in diabetic rats. Furthermore, foot process fusion was reduced, and ECM proliferation was alleviated in diabetic rats, indicating that renal structural damage was relieved by GSPE treatment. In addition, there were a large number of apoptosis in HK-2 cells cultured with high glucose *in vitro*. In contrast, the abnormal apoptosis of HK-2 cells was reduced in HG + GSPE incubation, indicating that diabetic renal injury was relieved by GSPE treatment. The above events suggested that GSPE treatment could protect the kidney from high glucose toxicity, thereby alleviating metabolic disorders, reducing renal structural damage, and improving the clinical symptoms of DKD. However, it is worth noting that GSPE also had hypoglycemic and weight-reducing effects in healthy rats. The mechanisms of GSPE affecting metabolism may be affecting the function of pancreatic β-cells, preventing the effect of a high-fat diet on pancreatic insulin secretion, regulating intestinal microflora, and upregulating intestinal GLP-1 receptor expression (Liu et al., 2020; Grau-Bové et al., 2021).

Accumulating evidence has demonstrated that hyperglycemia, high ROS production, and mitochondrial dysfunction are implicated in the development of DKD (Brownlee, 2005; Reidy et al., 2014; Jha et al., 2016). Among these factors, mitochondrial dysfunction is currently regarded as the key factor in the progression of DKD (Forbes and Thorburn, 2018; Qin et al., 2020). Mitochondrial biogenesis and dynamics are essential in sustaining mitochondrial homeostasis and quality (Bhargava and Schnellmann, 2017). Mitochondrial biogenesis, the generation of new mitochondria, is a complex process involving mtDNA replication and protein synthesis, which is regulated by mitochondrial and nuclear genomes (Scarpulla et al., 2012). PGC-1α is a master regulator of mitochondrial biogenesis, that coordinates the transcriptional machinery leading to increased mitochondrial mass, thus allowing the tissue to adapt to increased energetic demands (Lagouge et al., 2006). SIRT1 is a key regulator of energy and metabolic homeostasis (Rodgers et al., 2005). It is known that SIRT1 activates the PGC‐1α‐mediated transcription of nuclear and mitochondrial genes encoding proteins during mitochondrial proliferation and energy production (Popov, 2020). Subsequently, PGC-1α initiates the activation of NRF1, which then promotes TFAM activation (Hao et al., 2021). Once activated, TFAM translocates to the mitochondrial matrix and stimulates mtDNA replication and protein translation. On the other hand, mitochondrial dynamics, which contains two reverse processes, fission and fusion, directly contribute to the morphological changes in mitochondria. Previous studies have shown that hyperglycemia of diabetes directly promotes the fragmentation of mitochondria *via* activation of fission (Diaz-Morales et al., 2016). The fission process is mediated by a family of dynamin-related proteins (Drps), particularly Drp1 (Qin et al., 2019). Like fission, fusion controlled by MFN1 is critical for mitochondrial function (Youle and van der Bliek, 2012). Under TEM observation, normal mitochondria were usually in a fused state with a elongated rod shape. In contrast, the majority of mitochondria were spherical shape in the DM group, which was attributed to the interruption of mitochondrial fusion. In addition, we found that protein expression of mitochondrial biogenesis (SIRT1, PGC-1α, NRF1, TFAM) and fusion (MFN1) decreased *in vivo* and *in vitro*, while mitochondrial fission-related protein Drp1 increased. This suggested that disturbances in mitochondrial biogenesis and dynamics greatly promote mitochondrial dysfunction which eventually accelerates the progression of DKD. However, these changes were reversed after GSPE treatment. GSPE was demonstrated to affect physiological processes in renal tissues and HK-2 cells by enhancing mitochondrial biogenesis and weakening mitochondrial fission.

P66Shc is a member of the Shc protein family, which is an important regulatory protein involved in oxidative stress (Boengler et al., 2019). Recently, researchers discovered that the expression of p66Shc is significantly higher in diabetic patients than in non-diabetic patients (JZ et al., 2021). In the present study, we found that diabetic rats exhibited an increased level of p66Shc. The same phenomenon was observed in HK-2 cells cultured with high glucose, which were consistent with the results of previous literature. High glucose induces phosphorylation of p66shc, which enhances the translocation of p66Shc from the cytosol to the mitochondria. Once p66Shc has entered the mitochondria, the Cyto C release as well as hydrogen peroxide formation occurs (Miller et al., 2021). Subsequently, Δψm is down-regulated, which can trigger the opening of mitochondrial permeability pores, leading to mitochondrial swelling and promoting increased apoptosis (Giorgio et al., 2005). Meanwhile, excessive production of ROS reduced the activity of the mitochondrial respiratory chain and decreased ATP production, which would cause disturbances in mitochondrial structure and function (Spinazzi et al., 2012; Brand, 2016). Consistent with the above process, we demonstrated that inhibition of p66Shc was sufficient to raise mitochondrial function and reduce the incidence of apoptosis. However, GSPE abrogated the increase in p66Shc expression and reversed the above, suggesting p66Shc as a therapeutic target of GSPE.

It has been indicated that p66Shc can interfere with mitochondrial dynamics and biogenesis (Pérez et al., 2018) (Li et al., 2017) (Wu et al., 2019). Through transfection of si-p66Shc and over-p66Shc *in vitro*, our experiments confirmed that p66Shc regulated the efficiency of mitochondrial biogenesis by regulating the expression of SIRT1, PGC-1α, and its target genes NRF1 and TFAM. There was a significant drop in the protein level of MFN1 while the protein level of Drp1 increased when cells were overexpressed with p66Shc or stimulated by HG conditions. The dynamic balance of mitochondrial fission/fusion was disrupted. These results indicated that p66Shc reduced mitochondrial biogenesis and fusion in HK-2 cells, thereby promoting cell damage and apoptosis. These changes could be reversed by GSPE intervention. Therefore, GSPE may affect mitochondrial dynamics and biogenesis by negatively regulating p66Shc, to improve mitochondrial function and alleviate diabetes-induced apoptosis. This discovery has not been reported before.

## 5 Conclusion

In conclusion, our data showed for the first time that the nephroprotective effect of GSPE in DKD is mediated by suppression of p66Shc expression, and subsequently activation of mitochondrial biogenesis and inhibition of mitochondrial fission. That suggested p66Shc may represent an attractive target for GSPE in the treatment of DKD. Our data provide further support for the therapeutic efficacy of GSPE to promote mitochondrial function and provide benefits for DKD. We are optimistic that GSPE has great potential for further research.

## Data Availability

The original contributions presented in the study are included in the article/Supplementary Material, further inquiries can be directed to the corresponding authors.
