# Supplementary figures and images for: Grape seed proanthocyanidin extract targets p66Shc to regulate mitochondrial biogenesis and dynamics in diabetic kidney disease
Source: Front Pharmacol. 2023 Jan 6;13:1035755. doi: 10.3389/fphar.2022.1035755 (PMC9853208; doi:10.3389/fphar.2022.1035755)

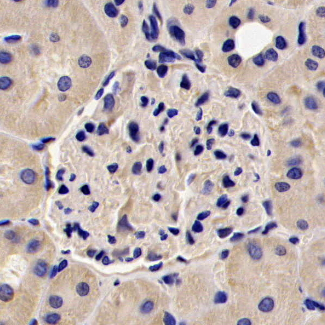

Supplement: Supplementary file 1 [file Image14.PNG]

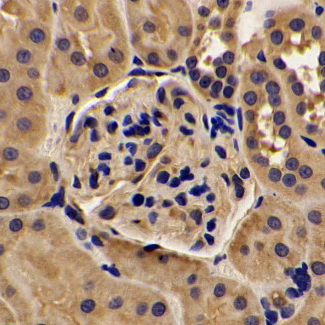

Supplement: Supplementary file 2 [file Image11.PNG]

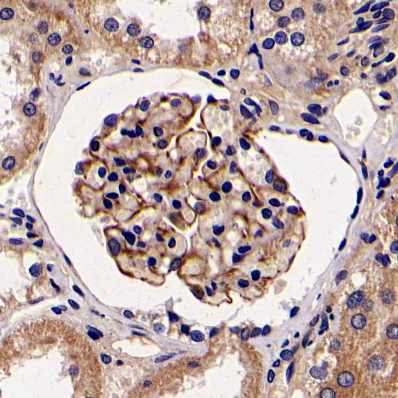

Supplement: Supplementary file 3 [file Image12.PNG]

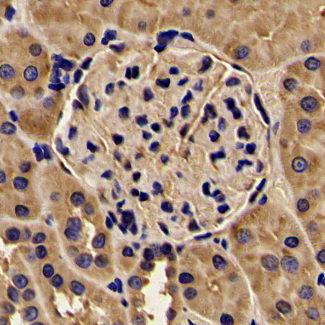

Supplement: Supplementary file 4 [file Image5.PNG]

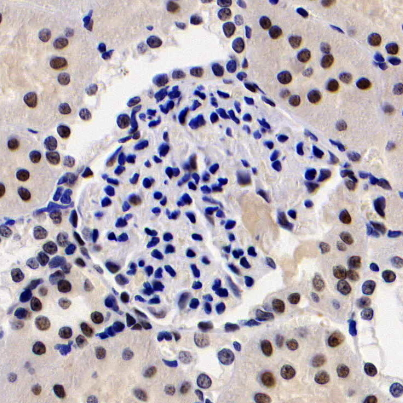

Supplement: Supplementary file 5 [file Image4.PNG]

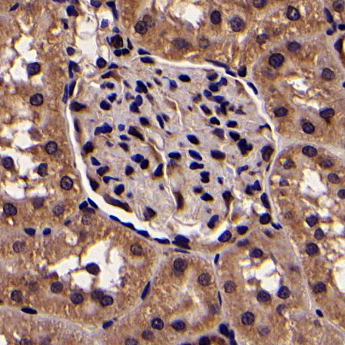

Supplement: Supplementary file 6 [file Image13.PNG]

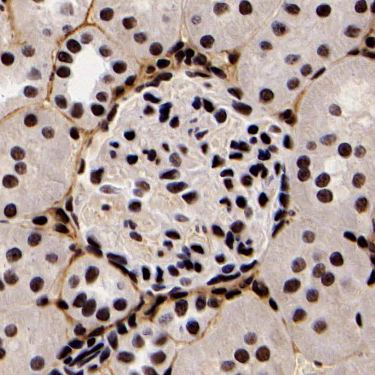

Supplement: Supplementary file 7 [file Image7.PNG]

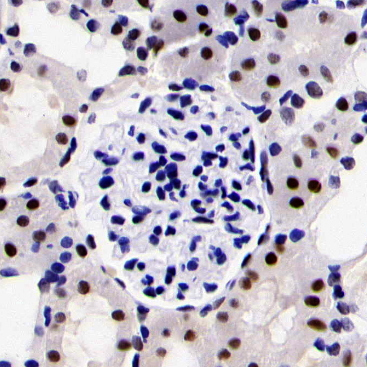

Supplement: Supplementary file 8 [file Image2.PNG]

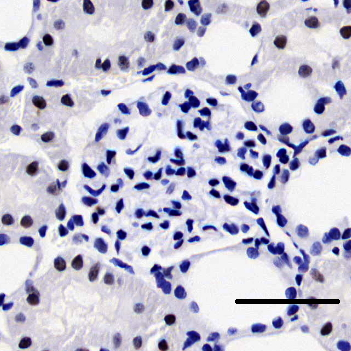

Supplement: Supplementary file 10 [file Image1.PNG]

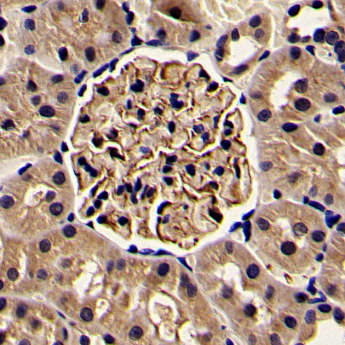

Supplement: Supplementary file 11 [file Image8.PNG]

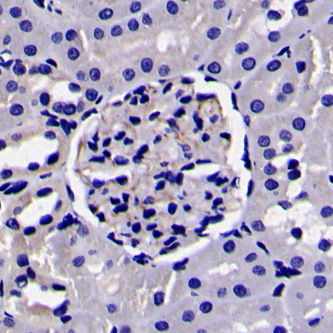

Supplement: Supplementary file 12 [file Image9.PNG]

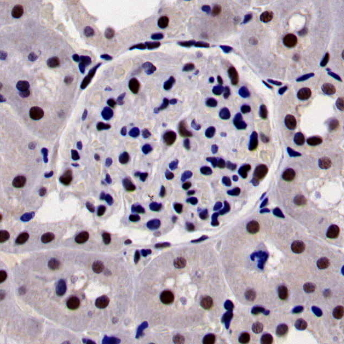

Supplement: Supplementary file 13 [file Image6.PNG]

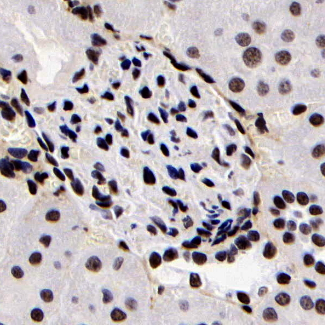

Supplement: Supplementary file 14 [file Image15.PNG]

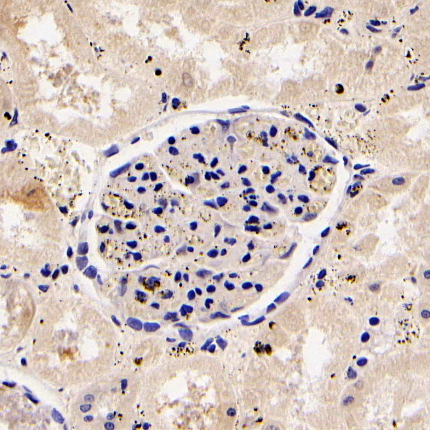

Supplement: Supplementary file 15 [file Image3.PNG]

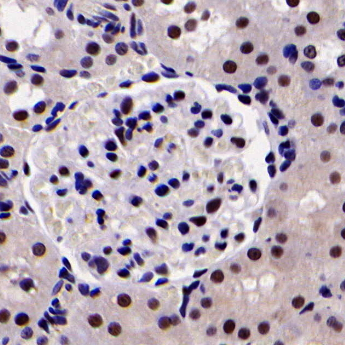

Supplement: Supplementary file 16 [file Image10.PNG]
